# Knowledge, Attitudes, and Practices Regarding Preconception Care Among Women in Saudi Arabia: A Cross-Sectional Study

**DOI:** 10.7759/cureus.71982

**Published:** 2024-10-21

**Authors:** Fawaz Edris, Mariah Almehmadi, Noor S Alharbi, Abeer Y Alalwani, Reham Hussien Alhazmi, Manar Salman Alharbi, Sara A Baghdadi, Iman Hamid Alenezi, Ahmed Baker A Alshaikh

**Affiliations:** 1 Obstetrics and Gynecology, Umm Al-Qura University, Makkah, SAU; 2 Medicine and Surgery, Umm Al-Qura University, Makkah, SAU; 3 Medicine, College of Medicine, Umm Al-Qura University, Makkah, SAU; 4 General Practice, Umm Al-Qura University, Makkah, SAU; 5 Obstetrics and Gynecology, Ministry of Health, Arar, SAU; 6 Obstetrics and Gynecology, College of Medicine, Jouf University, Sakaka, SAU

**Keywords:** attitudes, cross-sectional study, females, knowledge, practice, preconception care, saudi arabia

## Abstract

Background: Preconception care (PCC) aims to address health risks before conception for better pregnancy outcomes. Effective PCC starts before pregnancy and involves interventions to improve well-being and prevent adverse outcomes. Unintended pregnancies and pre-existing illnesses increase risks. Early provision of care is crucial, as fetal development can be affected early. Improving women's knowledge and access to PCC is essential for optimal maternal and child health in Saudi Arabia.

Methodology: A descriptive, cross-sectional study was conducted in Saudi Arabia to assess knowledge, attitudes, and practices regarding preconception care among women between the ages of 18 and 45 who live in Saudi Arabia, who matched the inclusion criteria. All women working in or studying in the medical field were excluded from the research. Data was collected using Google Forms (Google, Mountain View, CA) via social media apps. Data was cleaned in Microsoft Excel (Microsoft Corporation, Redmond, WA) and analyzed using IBM SPSS, version 29 (IBM Corp., Armonk, NY).

Results: Our study assessed awareness about PCC among 788 women. Age distribution revealed 24.9% (n=196) were 30-35 years old and 23.6% (n=186) were 40-45 years. Most were Saudi nationals (86.9%, n=685) and married (94.4%, n=744). University education was reported by 83.0% (n=654). Among notable findings, 79.1% disagreed or were neutral on PCC's necessity, and 78.6% supported free PCC. Barriers included fear of blood draws (68.7%) and negative family reactions (63.8%). Notably, 44.2% (n=348) demonstrated high knowledge and positive attitude towards PCC. Multivariate analysis indicated that marital status was significantly associated with high PCC knowledge (Exp(B)=1.956, p=0.049), while other sociodemographic factors were not associated with PCC knowledge.

Conclusion: Our study showed an adequate level of knowledge about PCC among women. However, there is a need for further improvement in PCC awareness among Saudi women. A significant number of women supported free PCC services. Barriers included fear of procedures and family reactions. Marital status was significantly associated with higher PCC knowledge, emphasizing targeted educational interventions.

## Introduction

Preconception care (PCC) is a set of interventions aiming to identify and address biological, behavioral, and social risks to a woman's health or pregnancy outcome through preventative measures and management [1,2]. However, to be most effective, these interventions should start before conception, as defined by the World Health Organization (WHO) [3].

PCC focuses on assisting individuals through services, treatments, support, and counselling in planning, playing an essential role in promoting short- and long-term well-being, disease prevention, and reducing the risk of poor pregnancy outcomes [4-6]. Evidence has shown that unintended and unplanned pregnancies are associated with an increased risk of poor pregnancy outcomes, with nearly half of all pregnancies in the developed countries being unintended, and approximately 52% of these women having at least one risk factor that could have a negative impact on pregnancy outcomes [7,8], because of pre-existing illnesses such as hypertension, diabetes, and infections [9]. More importantly, interventions given during the inter-conception period may minimize risks in further pregnancies [10,11]. The provision of health care is important, especially in high-income economies like Saudi Arabia.

Pregnancy-related risk factors can impact fetal development as early as 17 days after conception, before the initial prenatal checkup (6-10 weeks following conception) [12,13]. This can occur before the woman is aware of her pregnancy. Using multivitamins and folic acid before conception is a simple and effective method to improve pregnancy outcomes and fetal growth. Multivitamins may improve pregnancy outcomes by reducing the risk of preeclampsia, intrauterine growth restriction, preterm birth, placental dysfunction, and late fetal mortality. Folic acid supplementation is suggested before conception and early in pregnancy to avoid neural tube defects [14].

Preconception care is part of a larger healthcare ideal aimed at promoting healthy women, newborns, and families [15]. The first step in providing PCC is to understand women's access to health services and their awareness of preconception risk factors [16]. However, sometimes, awareness of preconception health is low, especially among those who have never had or are not planning a pregnancy [17,18]. This underlines the importance of improving knowledge regarding PCC to reduce risk factors for poor pregnancy outcomes while also optimizing mother and child health. Our goal was to study Saudi women's knowledge, attitudes, and behaviors related to preconception care, as well as identify barriers to accessing it.

## Materials and methods

Study design

A descriptive, cross-sectional study was conducted in Saudi Arabia to assess knowledge, attitudes, and practices in relation to preconception care among all women between the ages of 18 and 45 who live in Saudi Arabia, who matched the inclusion criteria. All women working in or studying in the medical field were excluded from the research.

Data collection

A validated questionnaire from a previous study [14], revised and translated into Arabic by a validated translator using the back-to-back technique, was distributed using Google Forms (Google, Mountain View, CA) via social media apps. The questionnaire was composed of four main components. The first section asked the participants about their demographic data, including age, nationality, marital status, education level, income, and employment status. The second section was about previous pregnancies and deliveries and a history of comorbidities. Participants were asked if they used birth control or assisted reproductive technology (ART), and if they had children, whether they had experienced any perinatal complications.

The third section assessed participants' knowledge of and attitudes towards preconception care, as well as women's social influences for seeking advice to attend PCC. The last section discussed women's belief in their capacity to execute the required behaviors prior to pregnancy and assessed women's understanding of risk factors for a safe pregnancy. A pilot research was conducted that included 20 married women to assess the reliability and validity of the questionnaire, as well as to measure the time required for its completion. To enhance clarity and understanding, complex medical terminology was omitted.

Statistical analysis

A comprehensive statistical analysis of the dataset was conducted, encompassing both descriptive and inferential methodologies. First, a descriptive analysis was conducted to summarize the demographic characteristics of the participants, which included age, gender, and other features. This provided an overview of the study population. Subsequently, inferential analyses such as Mann-Whitney U test and Kruskal-Wallis test were employed to examine the awareness score difference between variables while binary regression was used to find the predictor of high awareness. Statistical significance was established at a p-value of 0.05 or lower and a 95% confidence interval. All statistical analyses were executed using IBM SPSS, version 29.0.0 (IBM Corp., Armonk, NY).

Ethical consideration 

The research proposal received ethical approval from the Umm Al-Qura University Institutional Research Board/National Committee of Bioethics (registration no. HAPO-02-K-012). The questionnaire started with a clear statement that assured participants that their data would be anonymous and used solely for research purposes. Participants were given the option to agree or decline participation, and only those who agreed were included in the study. To maintain confidentiality and anonymity, each research participant was assigned a unique code number used solely for data analysis. Participation in the study was voluntary, and no incentives were offered. Furthermore, participants' identities were safeguarded in any published studies.

## Results

Our study included 788 women for the assessment of awareness about preconception care (Table 1). Notably, in terms of age distribution, 24.9% (n=196) were 30-35 years old, 23.6% (n=186) were 40-45 years of age, and smaller percentages belonged to other age groups. The majority were Saudi nationals (86.9%, n=685) and married (94.4%, n=744). Regarding education, 83.0% (n=654) were highly qualified. Income levels were fairly even, with 33.9% (n=267) earning 4000-10,000 Saudi riyal (SAR). Most participants were unemployed (57.9%, n=456), and 73.9% (n=582) resided in the Western Region. Chronic diseases were reported by 10.7% (n=84). Obstetrical history indicated 44.2% (n=348) had planned pregnancies, and 57.4% (n=452) had used birth control measures. Artificial insemination was utilized by 10.2% (n=80). Morbidities included premature births in 12.6% (n=100), neonatal ICU admissions in 9.5% (n=75), and low birth weight in 7.1% (n=56). Additionally, congenital malformations were reported by 3.0% (n=24) and low Apgar scores by 0.2% (n=2).

**Table 1:** Sociodemographic characteristics and past obstetrical parameters/history of patients

		Frequency (n=788)	Percentage
Age (years)	18-25	104	13.2
	25-30	149	18.9
	30-35	196	24.9
	35-40	153	19.4
	40-45	186	23.6
Nationality	Non-Saudi	103	13.1
	Saudi	685	86.9
Marital status	Divorced/widowed	44	5.6
	Married	744	94.4
Education	Primary/middle	15	1.9
	Secondary	119	15.1
	University education	654	83.0
Income	<4000 SAR	257	32.6
	4000-10,000 SAR	267	33.9
	>10,000 SAR	264	33.5
Employment	No	456	57.9
	Yes	332	42.1
Regions	Western Region	582	73.9
	Central Region	87	11.0
	Eastern Region	62	7.9
	Southern Region	43	5.5
	Northern Region	14	1.8
Chronic diseases (hypertension, diabetes mellitus, asthma)	No	704	89.3
	Yes	84	10.7
Parameters related to obstetric and maternal health services			
Planned previous pregnancy	Never pregnant	151	19.2
	No	289	36.7
	Yes	348	44.2
Ever taken birth control pills/used birth control devices?	Never pregnant	72	9.1
	No	264	33.5
	Yes	452	57.4
Ever gotten pregnant through artificial insemination/assisted devices	Never pregnant	103	13.1
	No	605	76.8
	Yes	80	10.2
Did you experience one or more of the following morbidities?	Premature birth (<37 weeks of gestation)	100	12.6
	Admission to neonatal ICU	75	9.5
	Low birth weight (<2500 grams)	56	7.1
	Congenital malformations	24	3.0
	Low Apgar score	2	0.2

Table 2 shows the assessment of knowledge and attitudes of participants regarding preconception care. A significant majority (79.1%, n=623) disagreed or were neutral about the idea that PCC before pregnancy is unnecessary, while 20.9% (n=165) agreed or totally agreed. The belief that PCC should be free for everyone planning to conceive was supported by 78.6% (n=619). Knowledge about healthy pregnancy through PCC was affirmed by 69.5% (n=548). Most participants (84.1%, n=663) expressed a willingness to attend free PCC counselling before pregnancy, and 71.7% (n=565) would go if it cost less than 55 SAR. A strong commitment to quitting smoking or drinking before pregnancy was shown by 84.6% (n=667). Access to monthly PCC advice and free tests before pregnancy was favored by 80.5% (n=634). Husband’s opinion was important to 77.7% (n=612) of participants, whereas family opinions were split almost evenly (50.4%, n=397). Friends’ opinions were less influential, with 84.0% (n=662) disagreeing or being neutral. Fear of social negative reactions on having a baby with health problems was a concern for 24.6% (n=194).

**Table 2:** Assessment of participants’ knowledge and attitudes regarding preconception care (PCC)

		Frequency (n=788)	Percentage
PCC before pregnancy is unnecessary.	Disagree/neutral	623	79.1
	Agree/totally agree	165	20.9
PCC should be free of charge for everyone planning to conceive.	Disagree/neutral	169	21.4
	Agree/totally agree	619	78.6
If you visit PCC, you know how to become pregnant in a healthy manner.	Disagree/neutral	240	30.5
	Agree/totally agree	548	69.5
If I have access to free PCC counselling before pregnancy, I would definitely go.	Disagree/neutral	125	15.9
	Agree/totally agree	663	84.1
If I have access to PCC counselling before pregnancy and it costs <55 SAR, I would definitely go.	Disagree/neutral	223	28.3
	Agree/totally agree	565	71.7
If I have to quit smoking and/or drinking before pregnancy, I will definitely quit.	Disagree/neutral	121	15.4
	Agree/totally agree	667	84.6
If I have access to PCC every month before pregnancy for advice and a free test, I will definitely go.	Disagree/neutral	154	19.5
	Agree/totally agree	634	80.5
My husband’s opinion is important to me.	Disagree/neutral	176	22.3
	Agree/totally agree	612	77.7
My family’s opinion is important to me.	Disagree/neutral	391	49.6
	Agree/totally agree	397	50.4
My friends’ opinion is important to me.	Disagree/neutral	662	84.0
	Agree/totally agree	126	16.0
I am afraid of social negative reactions if I have a baby with health problems.	Disagree/neutral	594	75.4
	Agree/totally agree	194	24.6

**Figure 1:**
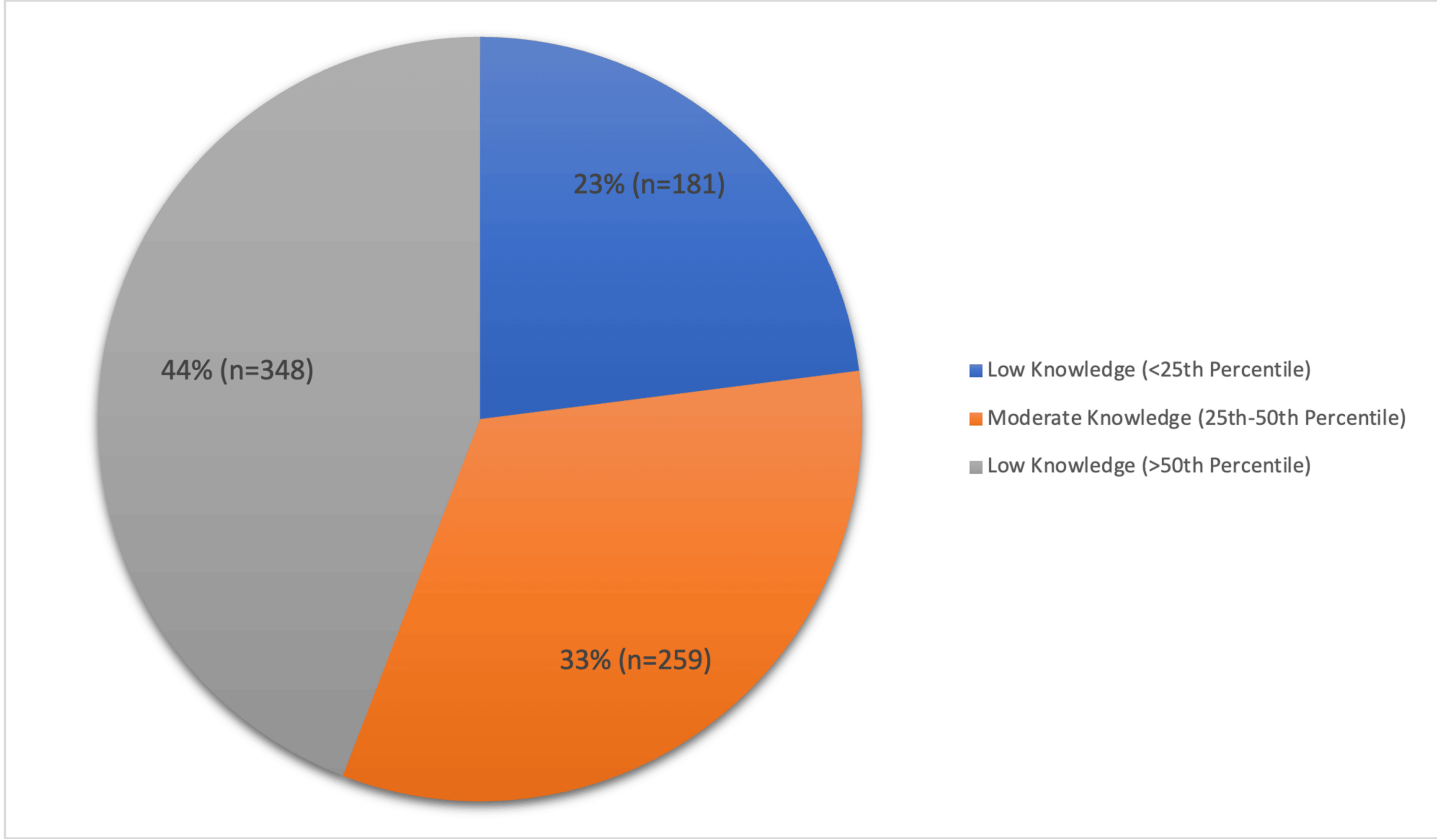
Overall knowledge and awareness about preconception care

Figure 1 shows the overall knowledge and awareness levels about preconception care. Low knowledge, defined as below the 25th percentile, was observed in 23.0% (n=181) of participants. Moderate knowledge, ranging between the 25th and 50th percentiles, was found in 32.9% (n=259) of the participants. The largest group, comprising 44.2% (n=348), demonstrated high knowledge, defined as above the 50th percentile.

Table 3 shows women's expectations and knowledge of risk factors for a healthy pregnancy. Regarding weight loss difficulty, 62.8% (n=495) found it very difficult or difficult, while 26.3% (n=207) found it very easy or easy. For smoking cessation, 33.6% (n=265) found it very easy or easy, but 28.0% (n=221) found it very difficult or difficult. Quitting alcohol was deemed very easy or easy by 32.5% (n=256) and very difficult or difficult by 19.7% (n=155). Daily folic acid intake was considered very easy or easy by 85.5% (n=674) and difficult by 10.2% (n=80). Attending PCC regularly was seen as very easy or easy by 81.3% (n=641) and difficult by 15.2% (n=120).

**Table 3:** Women’s self-expectations and knowledge of risk factors for a healthy pregnancy PCC, preconception care

		Frequency (n=788)	Percentage
If you are overweight, how difficult is it to lose weight?	Very easy/easy	207	26.3
	Very difficult/difficult	495	62.8
If you smoke, how difficult is it to quit?	Very easy/easy	265	33.6
	Very difficult/difficult	221	28.0
If you drink alcohol, how difficult is it to quit?	Very easy/easy	256	32.5
	Very difficult/difficult	155	19.7
How difficult is it to take folic acid daily?	Very easy/easy	674	85.5
	Very difficult/difficult	80	10.2
How difficult is it to attend PCC regularly (e.g., once per month) to seek advice or get information?	Very easy/easy	641	81.3
	Very difficult/difficult	120	15.2
Pregnancies close together are beneficial to the baby's health	False	629	79.8
	True	159	20.2
Smoking negatively affects fertility	False	38	4.8
	True	750	95.2
Underweight or overweight negatively affects fertility	False	94	11.9
	True	694	88.1
STDs should be treated before pregnancy	False	19	2.4
	True	769	97.6
All medications available in pharmacies are safe and can be used during pregnancy	False	727	92.3
	True	61	7.7
The best moment to start taking folic acid supplements is when you become pregnant	False	570	72.3
	True	218	27.7

Regarding risk factors, 79.8% (n=629) correctly identified that close pregnancies are not beneficial to the baby's health. A vast majority (95.2%, n=750) recognized that smoking negatively affects fertility, and 88.1% (n=694) acknowledged that being underweight or overweight affects fertility. Almost all participants (97.6%, n=769) understood the importance of treating STDs before pregnancy. Most (92.3%, n=727) knew not all medications are safe during pregnancy, and 72.3% (n=570) correctly noted that folic acid supplements should be started before pregnancy.

**Figure 2:**
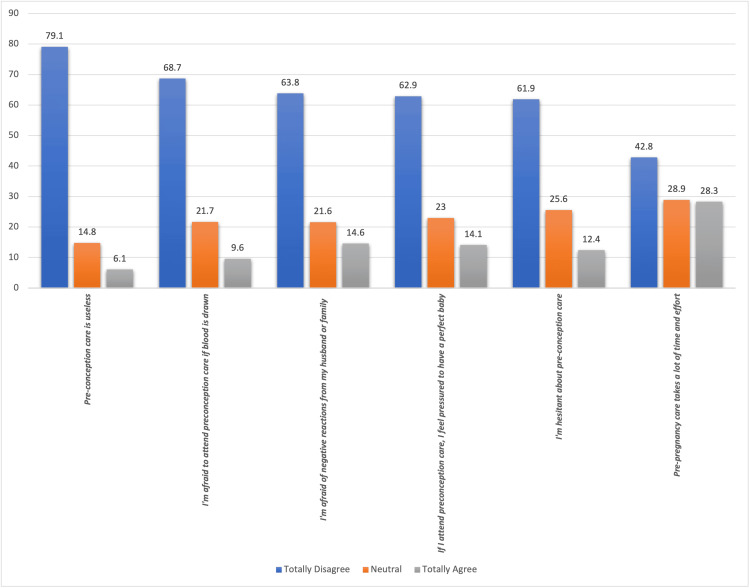
Barriers and obstacles that prevent Saudi women from accessing preconception care

Figure 2 shows the barriers and obstacles that prevent Saudi women from accessing preconception care, as perceived by participants. A notable finding is that 79.1% of participants totally disagreed with the notion that preconception care is useless. However, concerns about medical procedures were evident, with 68.7% expressing fear of attending PCC if blood is drawn. Additionally, 63.8% felt afraid of negative reactions from their husbands or family, while 62.9% felt pressured to have a perfect baby if they attended PCC. Other barriers included hesitancy about PCC (61.9%) and the perception that it requires significant time and effort (42.8%).

Table 4 explores the differences in knowledge scores regarding preconception care across various participant features. Age-wise, mean knowledge scores ranged from 37.66 (SD=7.95) for the 18-25 years group to 39.43 (SD=5.59) for the 25-30 years group, with no significant differences (p=0.790). Nationality did not significantly impact knowledge scores, with non-Saudis scoring 38.95 (SD=6.79) and Saudis scoring 38.74 (SD=6.83) (p=0.511). Marital status showed a marginally non-significant trend (p=0.061), where married women had a slightly higher mean score (38.90, SD=6.64) compared to divorced/widowed women (36.50, SD=9.20). Educational status did not significantly affect knowledge scores (p=0.216), with primary/middle education participants scoring 36.80 (SD=8.42), those with secondary education scoring 39.23 (SD=6.81), and highly qualified participants scoring 38.73 (SD=6.78). Employment status also showed no significant difference (p=0.544), with unemployed participants scoring 38.89 (SD=6.44) and employed participants 38.61 (SD=7.31). Income levels did not significantly affect scores (p=0.702), with scores of 38.70 (SD=6.90) for <4000 SAR, 39.11 (SD=6.31) for 4000-10,000 SAR, and 38.49 (SD=7.23) for >10,000 SAR. Regional differences were not significant (p=0.121), though mean scores varied slightly, with the Eastern Region scoring the highest (39.81, SD=6.09) and the Central Region, the lowest (37.45, SD=7.74). Finally, the presence of chronic comorbidities did not significantly impact knowledge scores (p=0.524), with those without comorbidities scoring 38.70 (SD=6.91) and those with comorbidities scoring 39.35 (SD=6.02).

**Table 4:** Knowledge score difference in relation to various participant features (univariate analysis) SAR, Saudi riyal

		N	Mean	SD	Sig. value
Age (years)	18-25	104	37.66	7.95	0.790
	25-30	149	39.42	5.58	
	30-35	196	39.25	5.84	
	35-40	153	38.64	7.31	
	40-45	186	38.45	7.49	
Nationality	Non-Saudi	103	38.95	6.79	0.511
	Saudi	685	38.74	6.82	
Marital status	Divorced/widowed	44	36.50	9.19	0.061
	Married	744	38.90	6.63	
Educational status	Primary/middle	15	36.80	8.41	0.216
	Secondary	119	39.22	6.80	
	Highly qualified	654	38.73	6.78	
Employment	No	456	38.88	6.44	0.544
	Yes	332	38.60	7.31	
Income	<4000 SAR	257	38.70	6.89	0.702
	4000-10,000 SAR	267	39.10	6.31	
	>10,000 SAR	264	38.48	7.22	
Region	Western Region	582	38.88	6.78	0.121
	Central Region	87	37.44	7.73	
	Eastern Region	62	39.80	6.08	
	Southern Region	43	38.83	6.42	
	Northern Region	14	37.28	5.64	
Chronic comorbidities (hypertension, diabetes mellitus, asthma)	No	704	38.70	6.90	0.524
	Yes	84	39.34	6.01	

Table 5 shows the results of multivariate analysis aimed to identify sociodemographic predictors associated with high knowledge levels regarding preconception care. Among the variables examined, only marital status showed a significant association with high knowledge levels, with married women having a significantly higher likelihood of possessing high knowledge about PCC (Exp(B)=1.956, p=.049, 95% CI: 1.003-3.814). However, other sociodemographic factors such as age (Exp(B)=.999, p=.991), nationality (Exp(B)=.883, p=.581), education level (Exp(B)=.956, p=.801), income (Exp(B)=.985, p=.880), employment status (Exp(B)=.968, p=.843), and the presence of chronic diseases (Exp(B)=1.308, p=.253) did not demonstrate significant associations with high knowledge levels.

**Table 5:** Adjusted sociodemographic predictors of high knowledge about preconception care (multivariate analysis)

	B	Sig.	Exp(B)	95% CI	
				Lower	Upper
Age	-.001	.991	.999	0.894	1.117
Marital status (married)	.671	.049	1.956	1.003	3.814
Nationality (Saudi)	-.124	.581	.883	0.569	1.372
Higher education	-.045	.801	.956	0.674	1.357
Higher income	-.016	.880	.985	0.804	1.205
Employment status (yes)	-.033	.843	.968	0.701	1.336
Suffer from chronic disease	.269	.253	1.308	0.826	2.072
Constant	-.618	.307	.539		

## Discussion

Preconception care aims to address health risks before conception for better pregnancy outcomes. According to Phalke et al. and Pimple and Ashturkar, preconception care is a set of interventions that aim to identify and modify biomedical, behavioral and social risks to women's health and pregnancy outcomes [19,20]. Effective PCC starts before pregnancy and involves interventions to improve well-being and prevent adverse outcomes. Withanage et al. showed that PPC delivered before conception can modify preconception risk factors and reduce the burden of adverse pregnancy outcomes such as low birth weight, spontaneous abortion, and preterm birth [21]. Unintended pregnancies and pre-existing illnesses increase pregnancy-related risks [22]. Early provision of care is crucial, as fetal development can be affected in the early stages of pregnancy. Improving women's knowledge and access to PCC is essential for optimal maternal and child health in Saudi Arabia. Our study examined Saudi women's awareness, knowledge, and attitudes around PCC, analyzing demographic factors, knowledge levels, barriers, and predictors, and comparing findings with the existing medical literature.

Notably, our study revealed a moderate (33%) to high (44%) level of awareness and knowledge regarding PCC among participants. Similarly, Boakye-Yiadom et al. showed that 23.5% had high knowledge levels in relation to preconception care [23]. A statistically significant majority recognized the importance of PCC and expressed willingness to attend counseling sessions and adopt healthy behaviors before pregnancy. A similar finding was observed by Fussi and Mandoura in their study that showed that preconception care was important for women of childbearing age and had a positive effect on pregnancy outcomes [24]. These findings are consistent with a previous research highlighting the growing awareness and efforts to increase awareness about PCC among women globally [25]. Besides the overall positive attitudes, some misconceptions and barriers persist, such as fears of medical procedures and societal pressures to have a perfect baby. Hence, addressing these misconceptions through targeted educational interventions and counseling services could further enhance awareness and uptake of PCC among Saudi women.

Comparing findings of our study with the previous medical literature provides valuable insights into the status of PCC awareness and knowledge in Saudi Arabia. While limited studies have specifically examined PCC awareness among Saudi women, broader research on maternal and reproductive health suggests similar trends. For instance, a study by Jehan et al. found that Saudi women have favorable attitudes towards antenatal care (89.7%), indicating a general receptiveness to preventive healthcare practices [26]. However, specific knowledge gaps and barriers to accessing care, such as cultural norms and lack of education, have been documented in previous studies. In a Kenya-based study, Okemo et al. highlighted some of the barriers to PCC that included women's lack of awareness of the risk factors and their impact on maternal and fetal outcomes, time constraints and healthcare providers' insufficient training and knowledge of PCC [27]. Moreover, according to the Kenya National Reproductive Health Strategy Report, some of the key challenges to the overall maternal and neonatal health service delivery were weaknesses in the health sector that negatively affect access to care and the various cultural and socioeconomic barriers to skilled care [28]. In addition, in a study by Fernandes et al., participants described various barriers such as an unsupportive organization and leadership, staff constraints, heavy workload, and resistance to change [29].

Moreover, the multivariate analysis in our study identified marital status as a significant predictor of high knowledge levels regarding PCC, with married women demonstrating a greater likelihood of possessing high knowledge. This finding underscores the importance of marital status as a sociodemographic factor influencing health-related behaviors and attitudes. However, Akinajo et al. did not show any association between awareness/knowledge about PCC and marital status [30]. While previous research suggests that marital status may influence healthcare utilization, particularly in maternal healthcare services, our study specifically aimed to explore the awareness of PCC among different marital groups. Studies have indicated that married women generally have higher utilization rates of maternal health care compared to their unmarried counterparts [31]. However, there is a notable gap in the literature regarding PCC awareness among unmarried women.

Implications

To enhance healthcare practice and policy, our study suggests implementing culturally sensitive educational campaigns to raise PCC awareness, training healthcare providers to address misconceptions, integrating PCC into maternal services, and initiating policies to tackle structural barriers like lack of healthcare financing and workforce training for equitable access to PCC services.

Limitations and future directions

Our study also had several limitations that should be kept in mind. The cross-sectional design precludes the cause-and-effect relationship in relation to the observed associations. Moreover, we need to conduct longitudinal studies, which will help to examine the temporal relationships between sociodemographic factors and PCC knowledge over time. Also, the sample of our study was drawn from specific regions in Saudi Arabia. This limits the generalizability of our study findings. Thus, we cannot apply these results to a broader population. Future researches should be done to include more diverse and representative samples to ensure the robustness and applicability of the findings.

## Conclusions

Our study contributes to our understanding of awareness, knowledge, and attitudes regarding preconception care among women in Saudi Arabia. While there is a moderate to high level of awareness and willingness to engage in PCC, misconceptions and barriers persist, highlighting the need for targeted educational interventions and policy initiatives to promote PCC uptake. By addressing these challenges and leveraging sociodemographic predictors such as marital status, healthcare providers and policymakers can play a pivotal role in improving maternal and child health outcomes in Saudi Arabia.
